# Associations between abdominal adiposity, body size and objectively measured physical activity in infants from Soweto, South Africa

**DOI:** 10.1007/s10995-022-03406-5

**Published:** 2022-05-25

**Authors:** Alessandra Prioreschi, Ken K Ong, Emanuella De Lucia Rolfe, Kate Westgate, Lisa K Micklesfield, Soren Brage

**Affiliations:** 1 SAMRC/WITS Developmental Pathways for Health Research Unit, Department of Paediatrics, School of Clinical Medicine, Faculty of Health Sciences, University of Witwatersrand, Johannesburg, South Africa; 2 MRC Epidemiology Unit, Institute of Metabolic Science, University of Cambridge, Cambridge, United Kingdom

**Keywords:** Toddler, Movement, Body composition, Accelerometry

## Abstract

**Objectives:**

Considering the importance of the early life period, in conjunction with the increasing prevalence of adiposity and insufficient physical activity already evident in early childhood, this study aimed to determine associations between abdominal adiposity, body size, and objectively measured physical activity in infancy.

**Methods:**

Infants (n = 138, aged 3–24 months) from Soweto, South Africa were recruited to this cross-sectional study. Visceral (VAT) and subcutaneous abdominal fat (SAT) were measured using ultrasound. Physical activity was assessed using accelerometry and analysed at the hourly level. Multilevel linear regression analyses were run with body composition exposures adjusted for age, sex, and length; models with VAT and SAT were also adjusted for total abdominal fat.

**Results:**

Mean (SD) age was 11.8 (7.6) months; 86% were normal weight, 7% were underweight and 7% overweight. In linear models, no body composition variable was significantly associated with physical activity. Physical activity was higher with each increasing length tertile (ANOVA p < 0.01); with a mean(95%CI) 29(60–60)mg in the lowest tertile, 39(71–71)mg in the middle tertile, and 50(81–82)mg in the highest tertile. Infants with normal weight had higher mean(95%CI) physical activity (40(70–80)mg) than underweight (34(73–85)mg, p = 0.01) or overweight infants (31(63–78)mg, ANOVA p < 0.01). When also adjusting for total abdominal fat, infants in the lowest SAT tertile had higher physical activity than those in the middle or highest SAT tertiles (p < 0.01).

**Conclusions:**

These findings lend support for higher physical activity as a marker of healthy growth in the first two years of life.

## Significance

While it is known that physical activity levels in infancy are often inadequate, and obesity is prevalent even in early childhood; there is limited and conflicting data on the relationship between physical activity and body composition in infancy. This is largely due to the nature of measures used. This study showed significant, non-linear relationships between infant objectively measured physical activity and adiposity assessed using abdominal ultrasound.

## Introduction

South Africa, much like the rest of the world, is experiencing a rapidly rising trend of increasing overweight and obesity (Shisana et al., 2014). At the same time, populations are becoming more sedentary and less physically active from a very young age (Shisana et al., 2014). Similar to international findings, the majority of South African children are not meeting physical activity recommendations, a problem which is worsening each year (Uys et al., 2016). This is of concern since these behaviours track into later childhood and adolescence (Benjamin-Neelon et al., 2020; Campbell et al., 2013; Carson, Tremblay, & Chastin, 2017). Determining correlates of physical activity in early life is therefore essential, in order to understand how to intervene effectively before poor trajectories are established. Furthermore, decreased physical activity levels in the early years may be indicative of poor health or malnutrition (Babirekere-Iriso et al., 2017; Yameogo et al., 2017), and a more thorough understanding of these relationships is thus important.

There is limited and mixed evidence for the relationship between body composition and physical activity in children under four years of age (Benjamin-Neelon et al., 2020; Carson, Lee, et al., 2017; Carson, Tremblay, et al., 2017; Collings et al., 2013; Timmons et al., 2012; Trost, Sirard, Dowda, Pfeiffer, & Pate, 2003). Longitudinal studies have examined this relationship, showing that physical activity in early childhood is associated with lower adiposity in later childhood (Berkowitz, Agras, Korner, Kraemer, & Zeanah, 1985; Moore, et al., 2003). How infant body composition associates with physical activity is less clear, largely due to the paucity of studies assessing physical activity in the first two years of life (Carson, Lee, et al., 2017; Ketcheson, Pitchford, Kwon, & Ulrich, 2017; Yameogo et al., 2017). In addition, most studies investigating these associations in the early years have used surrogate measures of adiposity such as body mass index (BMI) (Carson, Lee, et al., 2017) or skinfold thickness (Benjamin-Neelon et al., 2020), therefore limiting the conclusions that can be drawn on body composition per se. Measures of not only body size, but also body composition and adiposity deposition, such as ultrasound and CT or MRI imaging (De Lucia Rolfe et al., 2013; Suliga, 2009) may elucidate these relationships further.

It has been suggested that some studies may fail to observe associations between body composition and physical activity due to the common categorisation of physical activity data according to intensity thresholds, and the consequent focus placed on moderate to vigorous intensity physical activity, rather than examining physical activity as a continuum (Aadland, Kvalheim, Anderssen, Resaland, & Andersen, 2018; Collings et al., 2017). This may be particularly important in the first two years of life, during which time activities are intermittent and sporadic, and where hourly level data provides greater power to account for within-person diurnal variation (Prioreschi et al., 2017). Furthermore, hourly variations in the accumulation of daily physical activity during childhood and infancy have been shown (Faurholt-Jepsen et al., 2014; Prioreschi et al., 2017; Steele et al., 2010), and it is possible that these diurnal variations may affect the association between body composition and physical activity. Lastly, it is likely that the relationship between body composition and physical activity is bidirectional, particularly in this age group; whereby accumulating more activity may allow for better development and growth – the relationship most often explored. Conversely, better physically developed babies may be more likely to accumulate a greater volume of activity, either due to nourishment and/or development – and this relationship remains to be examined.

Considering the importance of this early life period in conjunction with the increasing prevalence of adiposity and insufficient physical activity already evident in early childhood, it is essential to determine whether there are associations between body composition and physical activity in the first two years of life. The aim of this study was therefore to determine the associations between abdominal adiposity (measured using ultrasound) and body size (BMI z-score and length) with objectively measured infant and toddler physical activity, while accounting for diurnal variation in physical activity.

## Methods:

### Participants and procedures:

Infants (n = 152) aged 3–24 months were recruited for this cross-sectional study performed at the SAMRC/Wits Developmental Pathways for Health Research Unit (DPHRU) at the Chris Hani Baragwanath Academic Hospital in Soweto, South Africa. Participants were excluded if they had been diagnosed with any developmental abnormalities that may impact normal movement or development. Mothers were asked to read and sign assent documents for their infant, and were free to withdraw from the study at any time. Ethical approval for this study was provided by the University of the Witwatersrand Human Research Ethics Committee (M150632). At recruitment, mothers were asked to report their date of birth, and their infant/toddler’s date of birth and gender.

### Measures:

#### Anthropometry and demographics:

All anthropometry measurements were taken twice and the average of the two values was used. Trained research staff measured length to the nearest 1 mm using an infantometer (Chasmors Ltd, UK), and weight to the nearest 0.1 kg using a digital scale (Dismed, USA). Weight, length, and BMI were converted to sex and age-specific z-scores according to the 2006 World Health Organisation (WHO) growth standards (de Onis, 2006) using the WHO Anthro software (“WHO Anthro for personal computers, version 3.2.2, 2011: Software for assessing growth and development of the world’s children.,“). Overweight was defined as a BMI z-score > 2, while underweight was defined as BMI z-score <-2.

### Abdominal adiposity:

In order to assess abdominal adiposity depots, infants were measured while lying quietly in the supine position using an ultrasound (GE, LOGIC e) with a standard curved array abdominal probe (4 C-RS–2–5 MHz 3 C-RS). The probe was positioned where the xiphoid line intercepts the waist circumference, and all images were captured during expiration. All measurements were performed twice by a trained operator according to previously validated techniques (De Lucia Rolfe et al., 2013), and were remeasured by a second trained operator (AP). Visceral adipose tissue (VAT) was measured with the probe in the longitudinal plane, and the distance between the peritoneum to the corpus of the lumbar vertebra was recorded. Subcutaneous adipose tissue (SAT) was measured with the probe in the vertical plane, and the distance between the linea alba and the cutaneous boundary was recorded. Where major discrepancies existed between the two measurements (±1SD of the mean difference between each operator’s measures, which equated to ±0.11 for SAT and ±0.23 for VAT), a third external operator (EDLR) re-measured the images and these values were then recorded and used. Lastly, 10% of all scans were re-measured by the external operator (EDLR) for further quality control. In all instances where discrepancies existed, the quality control measurement replaced the original measurement. If the image was deemed to be inaccurately captured or unusable, the value was rejected. If one image was usable but the second was not, the value for the usable image was used. Total abdominal thickness (TAT) was calculated as the sum of VAT and SAT.

### Physical activity:

Physical activity was objectively measured using an accelerometer (AX3, Axivity Ltd, Newcastle-upon-Tyne, UK) worn in a specially designed fabric band (Open Lab, Newcastle, UK). The design and feasibility of this infant band has been described previously (Prioreschi et al., 2018). Monitors were initialised to capture triaxial acceleration data at 100 Hz with a dynamic range of +-8 g and we requested that infants wore the monitors continuously for a 7-day period. Processing of the data, including non-wear detection and wear time criteria, have been described in detail previously (Brage et al., 2013; Pitchford, Ketcheson, Kwon, & Ulrich, 2017; Prioreschi et al., 2017; van Hees et al., 2014; van Hees et al., 2013; White, Westgate, Wareham, & Brage, 2016). The physical activity outcome, average vector magnitude of acceleration corrected for gravitational acceleration (HPFVM, in mg), was analysed at an hourly level; hours were excluded if any non-wear was detected within that hour. Participants were excluded if they did not provide at least 3 days with at least 15 h of data per day.

### Statistical analysis:

Data were stratified by sex and summarised as mean(SD) for parametric continuous data, n(%) for categorical data, and median(SEM) for nonparametric data. Two analytical approaches were used, whereby the exposure variables were first considered continuously, and then categorically. Firstly - pairwise correlations were run to determine significant correlates of average vector magnitude; then these correlates, as well as a priori determined confounders, were included in separate multilevel panel linear regression analyses with either BMI z-score or length (adjusted for age and sex), or TAT, VAT or SAT (adjusted for age, sex, and length) as exposures. Using substitution analysis, we then re-ran the VAT and SAT models also adjusting for TAT as has been suggested for this age group (Suliga, 2009); this describes the hypothetical effect of shifting one unit of SAT for one unit of VAT. All regression models were then re-run restricted to daytime hours only (between 7am and 7pm). Sensitivity analyses were conducted to determine potential modification effects by age (≤ 12 months (n = 93) or > 12 months (n = 45)).

Secondly - BMI z-score categories were used; while tertiles of length, SAT and VAT were created based on their residuals from models adjusted for age and sex (and for length in the case of SAT and VAT). After considering this main effect, SAT and VAT were further residualised for TAT. The hourly physical activity data were then used to plot diurnal distributions stratified by BMI categories and by tertiles of length SAT, and VAT. Differences in physical activity between the categories were tested using one way ANOVAs with Bonferroni post-hoc tests. Lastly, linear trends across these categories were tested using multilevel panel regressions. Lastly, the A p-value < 0.05 was considered to indicate statistical significance. All data were analysed using Stata version 13 (Stata Corp, College Station, TX) for Mac.

## Results:

Of the 152 infants recruited, 138 provided sufficient physical activity data for analysis. Of the 14 participants with insufficient data, 6 lost their measurement devices and 8 did not meet the wear time criteria. Of the 138 included participants, mean (SD) age was 11.8 (7.6) months, most (86%) were normal weight, 7% were underweight and 7% were overweight. Physical activity (mg) was higher in boys compared to girls; and boys were taller than girls, but there were no other sex differences detected (Table 1).

**Table 1:** Participant characteristics

	All (n = 138)	Boys (n = 74)	Girls (n = 64)	P-value
Age (months)	11.76 (7.59)	11.68 (7.59)	11.86 (7.80)	0.49
Length-for-age z-score	-0.92 (1.68)	-0.96 (1.46)	-0.88 (1.90)	0.16
Normal length (n(%))	106 (77)	58 (78)	48 (75)	0.02
Stunted (n(%))	32 (23)	16 (22)	16 (25)	
BMI z-score	0.25 (1.68)	0.23 (1.38)	0.28 (1.97)	0.41
Underweight (n(%))	10 (7)	4 (5)	6 (9)	0.81
Normal weight (n(%))	124 (86)	69 (88)	55 (83)	
Overweight (n(%))	10 (7)	5 (7)	5 (8)	
SAT (cm)	0.48 (0.08)	0.48 (0.08)	0.49 (0.09)	0.55
VAT (cm)	2.66 (0.66)	2.68 (0.52)	2.64 (0.83)	0.78
Average hourly movement (mg)	35.3 (0.5)	38.6 (0.8)	32.7 (0.7)	< 0.01

All values are mean(SD) except for the BMI categories which are n(%), and the hourly movement variable which is median(SEM)

### Linear associations

Length, BMI z-score, TAT, VAT, and SAT were not significantly linearly associated with physical activity (in adjusted models; Table 2). Findings were similar when physical activity was restricted to daytime hours only (Table 2).

**Table 2:** Associations between body composition variables and physical activity during the whole day and restricted to daytime hours

	Physical activity (mg) over 24 h				Physical activity (mg) in daytime hours			
Outcome variables	β coefficient	P value	95% CI		β coefficient	P value	95% CI	
Length (cm)	0.08	0.60	-0.23	0.40	0.03	0.91	-0.49	0.54
BMI z-score	-0.26	0.64	-1.38	0.85	-0.38	0.69	-2.28	1.51
TAT (cm)	-0.31	0.84	-3.21	2.59	-2.22	0.35	-6.84	2.40
SAT (cm)	6.80	0.41	-9.53	23.14	12.15	0.47	-20.56	44.86
VAT (cm)	-1.20	0.35	-3.72	1.32	-3.27	0.11	-7.27	0.73
SAT to VAT (cm)*	-5.08	0.63	-25.63	15.48	-12.15	0.47	-44.86	20.56

Results were derived from five separate multilevel regression models (for BMI z-score, length, total abdominal thickness, SAT, and VAT; and then for VAT vs. SAT substitution adjusted for total abdominal thickness)

Length models were controlled for age and sex

All VAT and SAT models were adjusted for age, sex, and length

* This model was further adjusted for TAT; i.e. representing effect of SAT to VAT substitution

Sensitivity analyses showed that findings with BMI z-score, VAT and SAT did not differ when stratifying the sample by age ≤ 12 months or > 12 months (data not shown). However, among younger infants aged ≤ 12 months, physical activity over the full 24 h was positively associated with length (β = 0.51, p < 0.01, 95% CI: 0.28–0.73) and TAT (β = 5.71, p = 0.03, 95% CI: 0.67–10.75).The association with length in this younger sub-group was even more pronounced with physical activity restricted to daytime hours only (β = 0.80, p < 0.01, 95% CI: 0.43–1.16). No association was seen in older infants aged > 12 months.

### Categorical associations

**Fig. 1:**
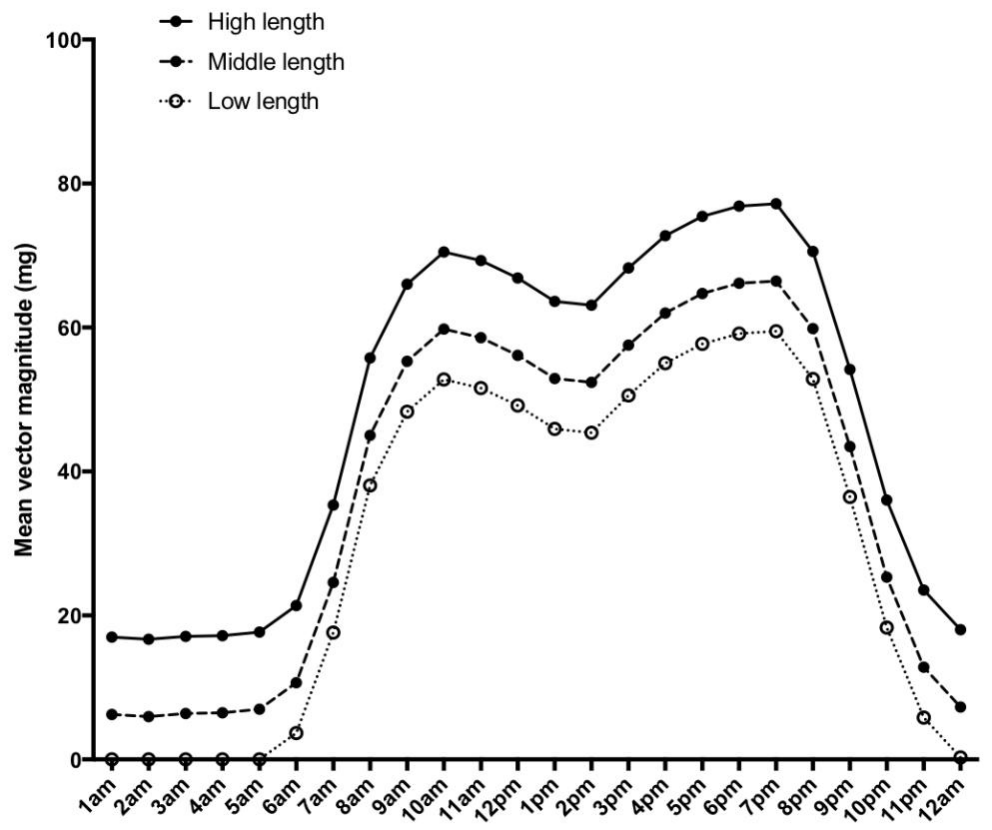
Mean infant physical activity (adjusted for infant age and sex) plotted by hour of the day, stratified by infant length tertiles

**Fig. 2:**
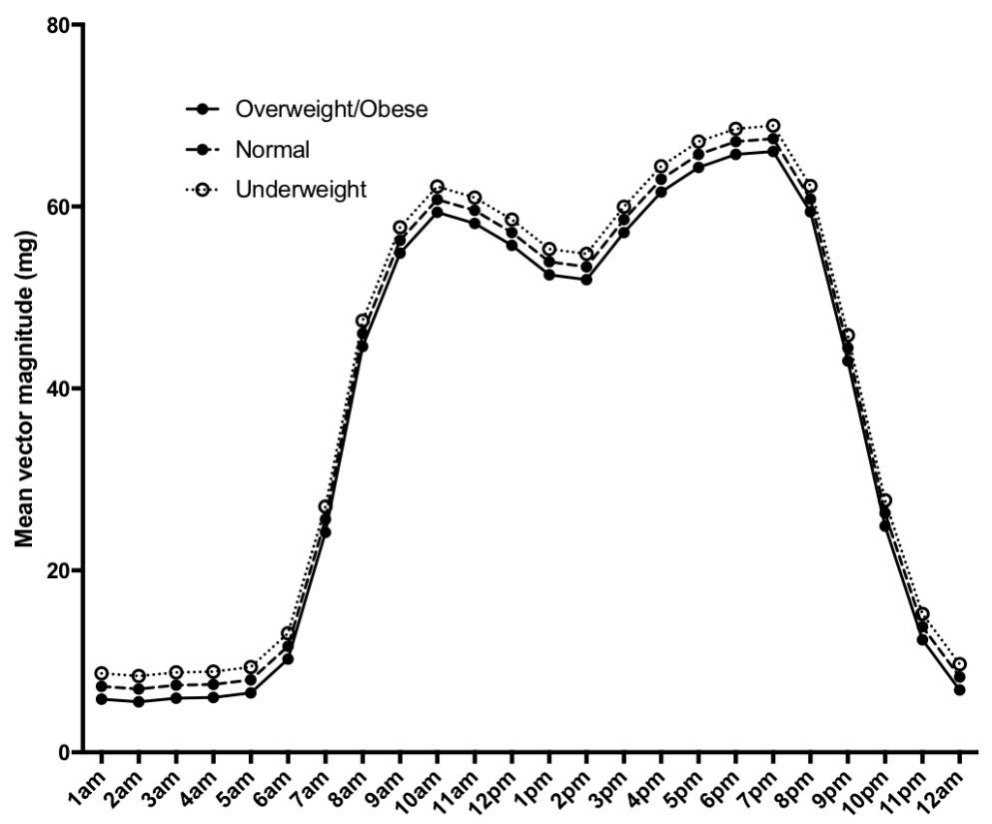
Mean infant physical activity (adjusted for infant age and sex) plotted by hour of the day, stratified by infant BMI categories

Mean physical activity was higher with each increasing length category (ANOVA p < 0.01); with a mean(95%CI) of 29(60–60)mg in the lowest tertile, 39(71–71)mg in the middle tertile, and 50(81–82)mg in the highest tertile (Fig. 1). By BMI category (Fig. 2), infants with normal weight had higher mean(95%CI) physical activity (40(70–80)mg) than underweight (34(73–85)mg, p = 0.01) or overweight infants (31(63–78)mg, ANOVA p < 0.01).

**Fig. 3:**
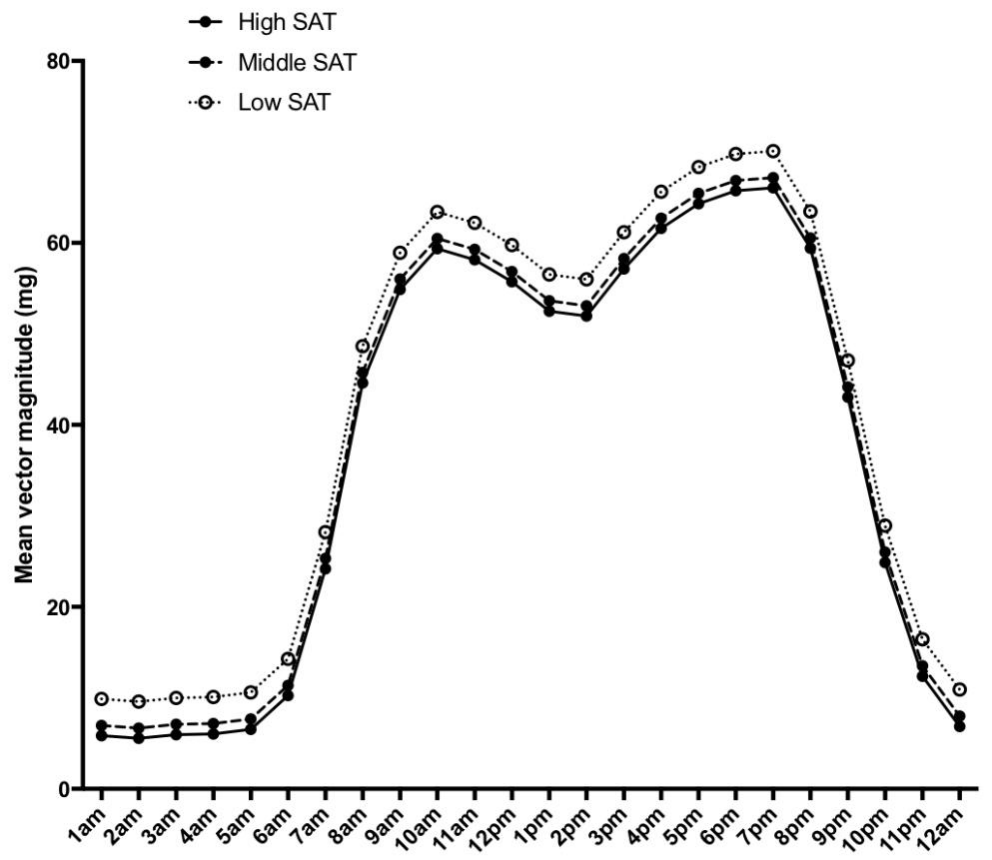
Mean infant physical activity (adjusted for infant age, sex, and length) plotted by hour of the day, stratified by infant SAT tertiles

**Fig. 4:**
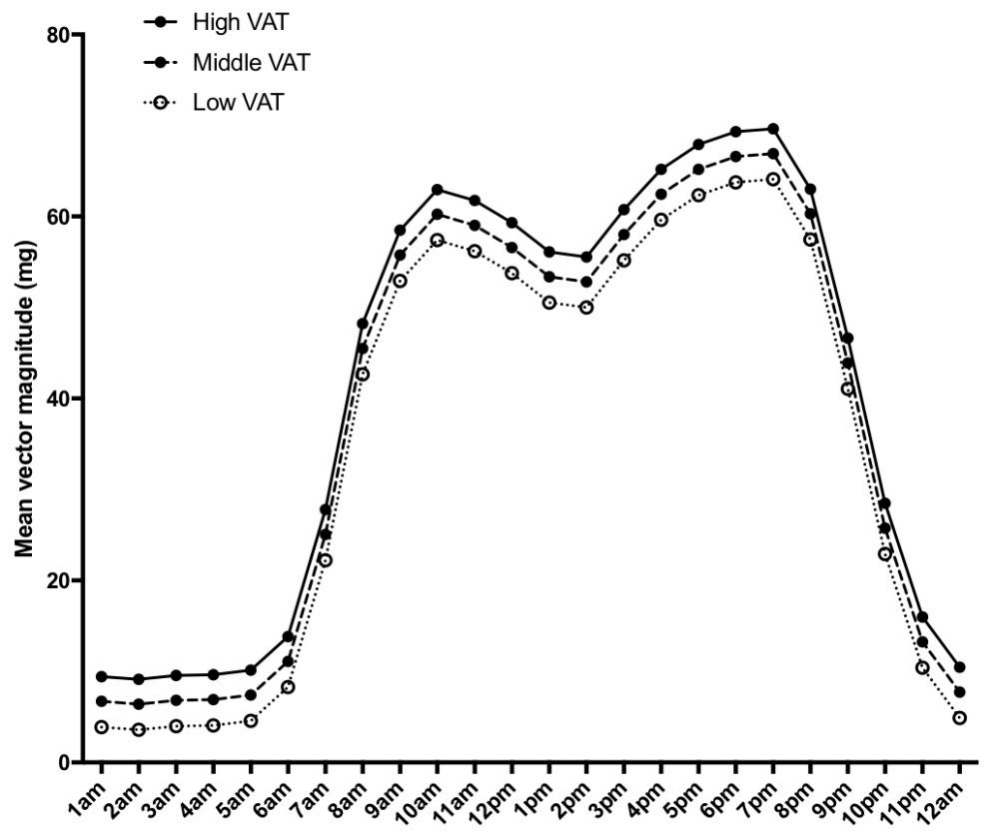
Mean infant physical activity (adjusted for infant age, sex, and length) plotted by hour of the day, stratified by VAT tertiles

Before adjusting for TAT, infants in the lowest SAT tertile had higher mean(95%CI) physical activity (44(0–0)mg) than infants in the middle (38(0–0)mg) or highest SAT tertile (35(1–1) mg), ANOVA p < 0.01 (Fig. 3). These associations with SAT tertiles persisted after adjusting for TAT. Conversely, before adjusting for TAT, infants in the lowest VAT tertile had lower physical activity (29(2–3)mg) than infants in the middle (42(3–3)mg) and highest VAT tertiles (42(3–3)mg), ANOVA p < 0.01 (Fig. 4). However, after adjusting for TAT, infants in the middle VAT tertile had higher physical activity (41(2–2)mg) than those in the lowest (34(3–3)mg) and highest VAT tertiles (38(3–3)mg), ANOVA p < 0.01.

In adjusted multilevel panel regression models, there was a positive association across length tertiles (β = 10.61, p < 0.01). There was no linear trend with physical activity across the BMI categories (β=-1.42, p = 0.57), yet there was a negative association across SAT tertiles (β=-4.43, p < 0.01 unadjusted for TAT; β=-4.46, p < 0.01 adjusted for TAT). While there was no linear trend across the VAT tertiles when adjusted for TAT (β = 1.10, p = 0.39); there was a positive association across VAT tertiles when unadjusted for TAT (β = 6.87, p < 0.01).

## Discussion

This study has explored the associations between infant abdominal fat depots and body size, with daily objectively measured physical activity, using imaging techniques and 24-hour accelerometry to more accurately measure these exposures and outcomes. Interestingly, while no significant associations were found between continuous adiposity or body size with physical activity; when categorising these exposures into tertiles the relationships became significant, indicating non-linear effects according to body size and abdominal fat depots. It appears from these results that infants who were thriving – i.e.: normal body weight, taller, lower SAT and ‘normal’ VAT (within the middle tertile), showed higher levels of physical activity.

Some of the relationships between fat deposition and physical activity exhibited a U-shaped relationship– such as BMI, and VAT tertiles when adjusted for TAT. Previous work in this age group has shown diurnal variations in physical activity intensity by age, sex, and developmental stage (Prioreschi et al., 2017). However, this is the first study we are aware of to show that diurnal variation in physical activity was differentially associated with high, middle and low infant body size and adiposity. It seems logical that bigger infants might accumulate more physical activity due to being better developed and able to produce bigger movements. However, it is interesting that that excessive body size (overweight and obese) appear to have a negative impact on physical activity. This implies that an optimal BMI allows higher physical activity levels compared to over- or under-weight infants. The present findings may provide some evidence for a beneficial relationship between optimal body composition and physical activity, even from a very young age. Similarly, when adjusted for TAT, a VAT within the middle tertile was associated with higher physical activity compared to the lowest or highest VAT tertiles. Typically, VAT has been associated with adverse metabolic and cardiovascular outcomes in children and adults (Staiano & Katzmarzyk, 2012; Suliga, 2009; Toro-Ramos, Paley, Pi-Sunyer, & Gallagher, 2015). However, there is limited research into the health consequences of VAT in infants (De Lucia Rolfe et al., 2013; Ferreira, da Silva Junior, Figueiroa, & Alves, 2014; Toro-Ramos et al., 2015).

On the other hand, length and SAT exhibited linear relationships with physical activity, where length was positively associated, and SAT inversely associated, with physical activity. Multiple studies in older children have shown that higher physical activity was associated with lower VAT, but not SAT (Suliga, 2009); however similar to our findings a study of children in a lower income setting in South America showed that higher physical activity was associated with lower SAT (as measured by skinfolds) (Urlacher & Kramer, 2018). Failure to reach growth potential (stunting) has consistently been associated with poor health outcomes in later life (Said-Mohamed, Pettifor, & Norris, 2017). In South Africa, stunting persists with nearly a third of children under the age of five years considered stunted according to the World Bank. Therefore, the positive association between length (adjusted for age and sex) and physical activity is indicative of infants who are thriving accumulating higher levels of activity. In order to further test these relationships, we repeated the length regression correcting for maternal height in order to account for a genetic influence; and the significance remained (data not shown). This indicates that these relationships were purely related to growth environments, and consequently are indicative of infants’ ability to thrive. Since both length, and physical activity during childhood have been related to improved outcomes (such as growth, cognitive and motor development, and cardio metabolic health) (Carson, Lee, et al., 2017; Said-Mohamed, Micklesfield, Pettifor, & Norris, 2015); it is encouraging that these two aspects of infant growth and development appear to be positively associated. Furthermore, the global focus on decreasing stunting (and improving body composition) in low to middle income countries such as South Africa may thus have a dual effect by concurrently improving infant movement behaviours through increasing physical activity.

To our knowledge this is one of the first studies to examine the association between body composition and physical activity in an infant population; and these cross sectional results must therefore be interpreted in the context of the dramatic and rapid changes in adiposity that occur during this period. However, a recent study that examined relationships between longitudinal physical activity and BMI and adiposity measured using skinfold thickness similarly did not find an association with linear outcomes, and attributed this to the use of skinfold thickness to estimate adiposity (Benjamin-Neelon et al., 2020). They did however find an association between physical activity and estimated central adiposity. It is important in the context of infant physical activity to consider theories related to the growth trade-offs that occur during the early years of life. Specifically, during critical periods of growth (i.e.: infancy) there is a limited amount of energy available for both growth and maintenance (Said-Mohamed et al., 2017), and focus is placed on brain and body growth during this period in order to maximise fitness. Therefore, if during this period, infants are exposed to undernutrition - which remains prevalent in the context of this study setting - they would preferentially allocate more energy to brain growth (Said-Mohamed et al., 2017). In line with this, while physical activity remains beneficial for motor and cognitive development (Carson, Lee, et al., 2017), and seems to be beneficially related to body composition in the current study; excessive energy expenditure during this time may also be detrimental for growth, forcing the infant to trade body growth for brain growth (Said-Mohamed et al., 2017). Given the importance of this period of life in setting up future body composition profiles (Young, Johnson, & Krebs, 2012), this improved understanding of how body size and adiposity are related to physical activity is crucial. Future research should include an assessment of nutritional status in order to account for the nutritional context of these findings, thus improving our understanding of the energy balance occurring during this period of life.

Studies examining the associations between physical activity and adiposity in the first few years of life do not show consistent findings, with many of these studies using proxy measures of adiposity (Carson, Lee, et al., 2017). It is possible that by using a more accurate measure of adiposity, we have been able to better determine associations between infant fat deposition. Furthermore, our objective measurement of physical activity allowed us to report diurnal variation over an extended period of time and representing the entire range of physical activity rather than categorised data, and has allowed us to examine differential diurnal relationships between physical activity and adiposity for the first time in this age group. Lastly, we have used a better measure of adiposity deposition, in conjunction with the commonly used BMI z-score, thus improving understanding of these relationships.

This study has several limitations. The cross sectional nature of the data limits our ability to determine causal relationships, and longitudinal growth and physical activity data in the first two years of life would greatly improve our understanding of these relationships. Secondly, the sample size was small, which limits the conclusions that can be drawn. The lack of information on the nutritional context of these findings means that we were unable to account for energy intake or undernutrition. We were also limited in our assumptions by the lack of birth outcome data, meaning we could not account for growth restriction in utero. Due to the nature of accelerometry and the limited methodological work that has done been done in this age group, we are not yet able to differentiate between infant initiated movement, and that initiated by a caregiver who may be carrying or moving the infant. Future methodological work is required to improve our ability to use accelerometry in infant populations.

In conclusion, using imaging measures of abdominal adiposity and an objective assessment of physical activity, this study shows that optimal body composition profiles were related to higher physical activity in infants in South Africa. Conversely, body composition profiles indicative of under- or overnutrition were associated with lower physical activity. These results provide the first insight into the effect that optimal growth may have on infants’ physical activity, and may be important for understanding how non-optimal growth may negatively impact health outcomes in children.

## Data Availability

De-identified data used in this manuscript are available upon request subject.

## List of abbreviations

BMI: body mass index.
SAT: Subcutaneous abdominal fat.
TAT: Total abdominal fat.
VAT: Visceral abdominal fat.
WHO: World Health Organisation.
